# Integrated SERS and Chemometric Characterization of Ultrasound‐Induced Quality Changes in Stevia‐Sweetened Aguaymanto (*Physalis peruviana* L.) Nectar

**DOI:** 10.1155/ijfo/2312352

**Published:** 2026-10-07

**Authors:** Betzabeth Brígida Huamán Estrada, Franklin Oré Areche, Peter Llimpe Perez, Norma Elvira Muguruza Crispin, Jorge Luis Rojas Paz, Fiorella Victoria Luperdi Rios, Jacqueline Roxana Reaño Rivera, Tania Jakeline Choque Rivera, Genaro Christian Pesantes Arriola, Carmen del Pilar Alvarez Quinteros

**Affiliations:** ^1^ Professional School of Agroindustrial Engineering Universidad Nacional José María Arguedas Andahuaylas Peru; ^2^ Professional School of Agroindustrial Engineering Universidad Nacional de Huancavelica Acobamba Peru; ^3^ Faculty of Bromatology and Nutrition Universidad Nacional José Faustino Sánchez Carrión Huacho Peru; ^4^ Department of Food Engineering Faculty of Fisheries and Food Engineering Universidad Nacional del Callao Callao Peru; ^5^ Professional School of Food Industries Engineering Universidad Nacional de Juliaca Juliaca Peru

**Keywords:** aguaymanto nectar, chemometric analysis, silver nanoparticles, surface-enhanced Raman spectroscopy, ultrasound processing

## Abstract

This study evaluated the molecular, physicochemical, antioxidant, color, and colloidal characteristics of stevia‐sweetened aguaymanto (*Physalis peruviana* L.) nectar under 10 processing‐formulation conditions combining ultrasound power (0–600 W), treatment time (0–15 min), and stevia concentration (0%–0.4%). Surface‐enhanced Raman spectroscopy (SERS) coupled with principal component analysis (PCA) was integrated with conventional physicochemical, antioxidant, CIELAB color, and particle‐characterization analyses. The citrate‐reduced AgNP substrate exhibited a surface‐plasmon‐resonance maximum at approximately 408 nm, a *Z*‐average diameter of 42.6 ± 6.3 nm, and a zeta potential of −37.8 ± 3.6 mV. Significant differences were observed among treatment combinations (*p* < 0.05), with T9 (600 W, 15 min, 0.4% stevia) exhibiting the most favorable overall instrumental profile. Particle size decreased from 980 nm in the untreated control to 270 nm in T9, whereas the absolute zeta potential increased from −18.2 to −37.8 mV and the PDI decreased from 0.49 to 0.21. T9 also showed the highest antioxidant, phenolic, flavonoid, and DCPIP‐reactive vitamin C responses. SERS revealed treatment‐dependent changes in characteristic bands at approximately 495, 1007, 1388, and 1613 cm^−1^, whereas PCA differentiated the treatment combinations according to their integrated molecular and biochemical profiles. Because ultrasound power, treatment duration, and stevia concentration were varied concurrently, the observed responses cannot be attributed independently to individual factors; therefore, T9 is considered the best performing combined condition rather than a statistically optimized formulation. Overall, integrating SERS and chemometrics with conventional quality measurements provides a promising analytical framework for evaluating processing‐induced changes in functional fruit beverages. Further factorial optimization, chromatographic validation, microbial and storage assessment, and sensory evaluation are required before industrial application.

## 1. Introduction

Aguaymanto is one of the functional fruits that have gained scientific and commercial interest due to their nutritional value and rich phytochemical profile [1]. The fruit is native to the Andean region of South America and is rich in vitamin C, carotenoids, polyphenols, flavonoids, withanolides, and dietary fiber, all of which are responsible for its antioxidant, anti‐inflammatory, antimicrobial, antidiabetic, and other health‐promoting properties [2, 3]. Its sweet‐acid flavor and beautiful yellow‐orange color make it a good raw material for juices and nectars as well as being nutritious. Industrial processing of aguaymanto, however, is still difficult since traditional thermal processes are prone to producing losses of antioxidant activity, vitamin C content, color quality, and sensory properties due to its heat sensitivity [4].

The increasing demand for healthier drinks has also drawn a quick pace toward the replacement of refined sugars with natural noncaloric sweeteners. Of these, the stevia berta (*Stevia rebaudiana* Bertoni) has been found to be one of the most promising alternatives due to the presence of steviol glycosides that are up to 200–300 times sweeter than sucrose and contain virtually no calories [5]. Besides their sweetening ability, stevia also has phenolics that have antioxidant properties, which can help preserve the oxidation‐sensitive components of the product during processing and storage [6, 7]. Thus, the use of stevia in the aguaymanto nectar is a promising strategy to create naturally sweetened functional beverages that can provide better nutritional value and lower sugar content.

Aguaymanto and stevia have high functional potential but the processing technology used has a strong impact on the quality of fruit beverages. Conventional thermal pasteurization is effective to create microbial stability, but it usually causes the degradation of thermolabile compounds, low vitamin content, and color and physicochemical instability. Such constraints have been a catalyst for the interest in developing new nonthermal technologies to provide preservation of nutritional and technological quality. Of these, high‐intensity ultrasound has attracted significant interest, as the cavitation effects created by the ultrasound generate localized micro high‐pressure environments, intense shear forces, micro streaming, and disruption of plant tissues, which improve mass transfer, release of intracellular compounds, reduce particle size, and increase dispersed system stability without causing such severe thermal damage as conventional processing [8, 9]. Resultingly, ultrasound‐assisted processing has emerged as a potential process option for the enhancement of good quality functional fruit beverages.

Most of the research has shown the advantages and benefits of ultrasound processing for fruit juices and plant‐based drinks, but most studies have been aimed at enhancing extraction efficiency or maintaining specific physicochemical properties [10, 11]. Molecular composition, antioxidant functionality, color properties, and colloidal stability have not been studied at the same time, which is relatively rare. These quality attributes are closely related to each other; therefore, assessing individual quality attributes does not give much insight into the mechanism of ultrasound induced quality improvement [8, 9, 12]. Thus, a more holistic understanding of processing‐induced changes in structure and function requires analytical strategies to be integrated.

Another drawback of existing studies is the use of traditional analytical methods (spectrophotometric antioxidant assays and physicochemical parameters) that do not provide sufficient information at the molecular level. Recently, a new rapid, sensitive, and nondestructive technique, known as surface‐enhanced Raman spectroscopy (SERS), has been developed that can provide molecular fingerprints in a simple manner with minimal sample preparation. SERS, in conjunction with chemometric techniques like principal component analysis (PCA), facilitates fast discrimination of food samples, detection of characteristic molecular markers, and correlation of the spectral data with biochemical composition [13, 14]. Although these benefits have been observed, the use of these beverages in naturally sweetened fruit beverages that are treated with ultrasound is still restricted.

Other factors, such as color stability and colloidal properties, are also crucial factors affecting consumer acceptance and product quality. Although the CIELAB color coordinates, particle size distribution, zeta potential, and polydispersity index (PDI) are readily measured, the data are often used as stand‐alone parameters and therefore not fully understood what the relationship is between the stability of pigment dispersions, the size of the particles and the molecular composition. The use of color analysis, particle characterization, and multivariate statistical techniques has been demonstrated to offer a more comprehensive view of the structural modifications that take place during food processing. However, such integrated evaluations are not frequently found for fruit beverages treated with ultrasound, especially for natural sweeteners.

Although ultrasound technology is increasingly being used in fruit processing, there are still several significant areas of knowledge that need to be addressed. The antioxidant activity, physicochemical properties, color characteristics, particle stability, and molecular fingerprint have been separately evaluated in the previous studies; however, in one study, these complementary datasets have been brought together to take a comprehensive picture of the phenomenon and to explain the mechanism that improves the quality. Moreover, though SERS is a very useful method for rapid characterization of food, it has not been widely used to analyze functional beverages with natural sweetness and ultrasound treatment. Therefore, the interactions between molecular composition, antioxidant activity, color changes, and colloidal stability over ultrasound processing remain not well understood and limit the development of more comprehensive strategies for quality evaluation of functional beverages.

To address these limitations, the present study employed an integrated analytical strategy combining SERS with comprehensive physicochemical characterization, antioxidant profiling, CIELAB color analysis, particle characterization, and multivariate chemometric techniques. Rather than evaluating individual quality attributes separately, the resulting datasets were integrated using PCA, correlation analysis, and standardized visualization approaches to establish relationships among molecular fingerprints, physicochemical properties, antioxidant activity, color characteristics, and colloidal stability. This multidimensional approach provides a more comprehensive understanding of ultrasound‐induced quality modifications than conventional single‐parameter analyses.

The central hypothesis of this study was that the combined application of ultrasound processing and stevia incorporation would improve the molecular, physicochemical, antioxidant, color, and colloidal characteristics of aguaymanto nectar through cavitation‐induced structural modification, enhanced release of intracellular bioactive compounds, particle‐size reduction, and improved electrostatic stabilization. The novelty of this research lies in integrating SERS molecular fingerprinting with comprehensive antioxidant evaluation, advanced color characterization, particle stability assessment, and chemometric interpretation within a single analytical framework for stevia‐sweetened aguaymanto nectar. To the best of our knowledge, few previous studies have combined these complementary analytical approaches to evaluate quality changes in ultrasound‐treated functional fruit beverages.

Therefore, the objective of this study was to evaluate the effects of different ultrasound‐processing conditions, generated by varying ultrasound power, treatment time, and stevia concentration, on the molecular, physicochemical, antioxidant, color, and colloidal characteristics of stevia‐sweetened aguaymanto (*Physalis peruviana* L.) nectar using an integrated analytical platform combining SERS, conventional quality analyses, and multivariate chemometric techniques.

## 2. Materials and Methods

### 2.1. Materials

Fresh aguaymanto (*P*. *peruviana* L.) fruits at commercial maturity were obtained from a local producer in Peru and transported to the laboratory under refrigerated conditions (4^°^C ± 1^°^C). Fruits were manually sorted to remove damage, immature, or diseased samples before processing. Food‐grade stevia powder (≥ 98% rebaudioside A purity) was used as the natural sweetener. All analytical‐grade reagents, including 2,2‐diphenyl‐1‐picrylhydrazyl (DPPH), 2,2 ^′^‐azinobis‐(3‐ethylbenzothiazoline‐6‐sulfonic acid) (ABTS), Folin–Ciocalteu reagent, gallic acid, quercetin, Trolox, potassium persulfate, ferric chloride, 2,4,6‐tripyridyl‐s‐triazine (TPTZ), metaphosphoric acid, and other chemicals used for physicochemical and antioxidant analyses, were purchased from Sigma‐Aldrich (St. Louis, Missouri, United States). Ultrapure deionized water (18.2 M*Ω* cm) was used throughout the experiments.

### 2.2. Preparation of Aguaymanto Nectar

Fresh aguaymanto fruits at commercial maturity were washed thoroughly under running potable water, sanitized by immersion in a sodium hypochlorite solution (100 mg L^−1^ available chlorine) for 2 min, rinsed with distilled water, and drained. The fruits were manually cut, and the pulp was separated from the seeds using a laboratory pulper. The nectar was prepared by blending aguaymanto pulp with distilled water according to the formulation shown in Table 1. Food‐grade stevia powder (≥ 98% rebaudioside A) was incorporated at the concentrations specified in the experimental design and homogenized for 2 min using a high‐speed homogenizer (Ultra‐Turrax T25, IKA‐Werke GmbH & Co. KG, Staufen, Germany) operating at 10,000 rpm. The prepared nectar was transferred into sterile borosilicate glass beakers and immediately subjected to ultrasound treatment. Samples not processed immediately were maintained below 10°C to minimize enzymatic degradation and oxidation before sonication. The preparation procedure was adapted from previous studies on ultrasound‐assisted processing of fruit beverages with minor modifications [8, 9, 12].

**Table 1:** Experimental treatments.

Treatment	Ultrasound power (W)	Time (min)	Stevia (%)
T1	0	0	0.0
T2	200	5	0.2
T3	200	10	0.4
T4	400	5	0.2
T5	400	10	0.4
T6	400	15	0.0
T7	600	5	0.4
T8	600	10	0.2
T9	600	15	0.4
T10	400	10	0.2

### 2.3. Experimental Design and Ultrasound Treatment

The experiment consisted of 10 predefined treatment combinations established as an exploratory screening design to compare practically relevant processing conditions for stevia‐sweetened aguaymanto nectar. Ultrasound power ranged from 0 to 600 W, treatment time from 0 to 15 min, and stevia concentration from 0 to 0.4% (Table 1). The treatment combinations were selected to cover low, intermediate, and high processing conditions while limiting the number of experimental runs required for the integrated physicochemical, antioxidant, spectroscopic, color, and colloidal analyses. The design was therefore intended to identify promising combined treatment conditions rather than to estimate the independent effects of ultrasound power, processing time, or stevia concentration.

Ultrasound processing was performed using a VCX 750 ultrasonic processor (Sonics & Materials Inc., Newtown, Connecticut, United States) equipped with a 13‐mm titanium alloy probe, operating at a fixed frequency of 20 kHz and a maximum nominal output power of 750 W. Approximately 200 mL of nectar was transferred into a 250‐mL borosilicate glass beaker, and the probe was immersed approximately 2 cm below the sample surface. Continuous magnetic stirring was applied throughout sonication to ensure homogeneous energy distribution within the sample. The ultrasonic processor was operated in continuous mode, and the applied power levels (200, 400, and 600 W) were selected according to the experimental design. To minimize temperature increases caused by acoustic cavitation, the beaker was immersed in an ice‐water bath, and the sample temperature was maintained below 30^°^C ± 2^°^C during processing. Following sonication, the samples were immediately cooled to room temperature and stored at 4°C. All physicochemical, antioxidant, SERS, color, and particle characterization analyses were completed within 24 h after ultrasound treatment.

Because ultrasound power, processing time, and stevia concentration were varied concurrently across the predefined treatments, the three factors were not statistically orthogonal. Consequently, their individual main effects and interaction effects cannot be separated from the present dataset. Statistical inference was therefore restricted to comparisons among the 10 complete treatment combinations. Accordingly, T9 and the other treatments should be interpreted as integrated processing‐formulation conditions rather than as evidence of an independent effect of any single processing variable. A full factorial or response‐surface design incorporating matched ultrasound‐ and stevia‐only controls will be required in subsequent studies to quantify the independent and interactive contributions of these factors.

### 2.4. SERS Analysis

SERS was employed to characterize the molecular modifications induced by ultrasound processing in stevia‐sweetened aguaymanto nectar. Approximately 1 mL of nectar was mixed with an equal volume of freshly prepared silver nanoparticle (AgNP) colloid and gently vortexed for 30 s to ensure uniform mixing before being transferred into a quartz cuvette for spectral acquisition. Raman spectra were collected using a DXR3 Raman Microscope (Thermo Fisher Scientific, Waltham, Massachusetts, United States) equipped with a 785‐nm diode laser operating at 100 mW. Spectra were recorded over the spectral range of 400–1800 cm^−1^ using a 50× objective lens with a spectral resolution of approximately 4 cm^−1^. An integration time of 10 s with three accumulations per spectrum was applied to improve the signal‐to‐noise ratio. Three independent spectra were acquired from different locations of each sample to minimize sampling heterogeneity and improve spectral reproducibility.

Before spectral acquisition, the Raman spectrometer was calibrated using the characteristic 520.7 cm^−1^ Raman band of a silicon reference standard to ensure wavelength accuracy. Raw spectra were preprocessed using OMNIC Paradigm software (Thermo Fisher Scientific, United States) through sequential cosmic‐ray removal, adaptive polynomial baseline correction, Butterworth smoothing, vector normalization, and peak alignment prior to chemometric analysis. Characteristic Raman bands were assigned according to published spectral libraries for plant‐derived bioactive compounds and subsequently used for PCA and Pearson correlation analysis to evaluate relationships between molecular fingerprints and conventional quality attributes.

#### 2.4.1. Preparation and Characterization of AgNPs

AgNPs used as the SERS substrate were prepared by citrate reduction according to Lee and Meisel [15]. An aqueous AgNO_3_ solution (1 mM) was brought to boiling under continuous magnetic stirring, followed by dropwise addition of trisodium citrate solution (1%, w/v). The mixture was maintained at boiling for approximately 15 min until a pale‐yellow/gray colloidal suspension developed. After cooling to room temperature, the AgNP colloid was stored in amber glass containers at 4°C until analysis.

Optical characterization was performed using a UV‐1800 UV–Vis spectrophotometer (Shimadzu Corporation, Kyoto, Japan) over 300–700 nm, with AgNP formation evaluated from the characteristic surface‐plasmon‐resonance band in the 400–420 nm region. Hydrodynamic diameter, PDI, and zeta potential were determined at 25^°^C ± 1^°^C using a Zetasizer Nano ZS90 (Malvern Panalytical Ltd., Malvern, United Kingdom) by dynamic and electrophoretic light scattering. Measurements were performed in triplicate and expressed as mean ± standard deviation (SD).

Particle morphology and primary size were further characterized by transmission electron microscopy (TEM). Approximately 5 *μ*L of appropriately diluted AgNP suspension was deposited onto a Formvar/carbon‐coated 200‐mesh copper grid and air‐dried. Micrographs were acquired using a JEOL JEM‐2100 transmission electron microscope (JEOL Ltd., Tokyo, Japan) operated at 200 kV. Primary particle diameters were quantified from representative micrographs using ImageJ (National Institutes of Health, Bethesda, Maryland, United States), with at least 200 nanoparticles measured to obtain particle‐size distribution. This complementary characterization enabled assessment of the optical response, hydrodynamic behavior, surface charge, morphology, and primary size of the AgNP substrate used for SERS analysis.

### 2.5. Physicochemical Analysis

The physicochemical properties of ultrasound‐treated aguaymanto nectar were determined according to the AOAC Official Methods of Analysis [16]. The pH was measured using a calibrated digital pH meter (SevenCompact S220, Mettler Toledo, Greifensee, Switzerland) after calibration with standard buffer solutions (pH 4.00 and 7.00) before each analytical session. Total soluble solids (TSS) were determined at 20^°^C ± 1^°^C using a digital refractometer (PAL‐1, Atago Co. Ltd., Tokyo, Japan) and expressed as °Brix. The refractometer was calibrated with distilled water before each measurement. Titratable acidity (TA) was determined by titration with standardized 0.1 N NaOH to an endpoint of pH 8.2 and expressed as percentage citric acid equivalents (%).

Apparent viscosity was measured at 25^°^C ± 1^°^C using a Brookfield DV2T rotational viscometer (Brookfield Engineering Laboratories, Middleboro, Massachusetts, United States) equipped with an appropriate spindle operating at a constant rotational speed. Prior to analysis, nectar samples were equilibrated at the measurement temperature for 5 min to ensure thermal stability. Turbidity was determined spectrophotometrically at 660 nm using a UV‐1800 UV–Vis spectrophotometer (Shimadzu Corporation, Kyoto, Japan) and expressed as nephelometric turbidity units (NTU) following the method described by Tiwari [12]. All physicochemical measurements were performed in triplicate, and the results were expressed as mean ± SD.

### 2.6. Determination of Antioxidant Activity and Bioactive Compounds

The antioxidant capacity of ultrasound‐treated aguaymanto nectar was evaluated using three complementary assays: 2,2‐diphenyl‐1‐picrylhydrazyl (DPPH) radical‐scavenging activity [17], 2,2 ^′^‐azinobis‐(3‐ethylbenzothiazoline‐6‐sulfonic acid) (ABTS) radical‐cation decolorization assay [18], and ferric reducing antioxidant power (FRAP) assay [19]. Appropriate aliquots of nectar were reacted with the respective assay reagents according to the referenced procedures, and absorbance was measured using a UV‐1800 UV–Vis spectrophotometer (Shimadzu Corporation, Kyoto, Japan). Trolox was used as the calibration standard for DPPH and ABTS, and the results were expressed as *μ*mol Trolox equivalents (TE) g^−1^ fresh weight. FRAP results were expressed as *μ*mol Fe^2+^ g^−1^ fresh weight.

Total phenolic content (TPC) was determined using the Folin–Ciocalteu colorimetric method described by Singleton et al. [20], with gallic acid as the calibration standard, and expressed as mg gallic acid equivalents (GAE) g^−1^ fresh weight. Total flavonoid content (TFC) was determined using the aluminum chloride colorimetric method of Chang et al. [21], with quercetin as the calibration standard, and expressed as mg quercetin equivalents (QE) g^−1^ fresh weight.

Vitamin C was determined using the 2,6‐dichlorophenolindophenol (DCPIP) method according to AOAC International [16] and expressed as mg ascorbic acid equivalents per 100 g fresh weight. Because the DCPIP assay is based on a redox reaction rather than selective chromatographic separation of L‐ascorbic acid, potential contributions from other reducing constituents present in the fruit matrix cannot be excluded. All antioxidants and bioactive‐compound analyses were performed in triplicate, and the results are reported as mean ± SD.

### 2.7. Color Measurement and Particle Characterization

Color measurements were performed using a portable colorimeter (CR‐400, Konica Minolta Sensing Inc., Osaka, Japan) calibrated against a standard white reference plate before each analytical session. The CIELAB color coordinates (L*, a*, and b*) were recorded directly according to the Commission Internationale de l′Éclairage (CIE) color system under standard illuminant D65 with a 10° standard observer. From these primary color coordinates, the total color difference (*Δ*E), chroma (C*), hue angle (h°), whiteness index (WI), browning index (BI), and yellowness index (YI) were calculated using standard CIELAB equations described by Pathare et al. [22]. Three independent measurements were obtained for each sample, and the average values were used for statistical analysis.

Particle size distribution, zeta potential, and PDI were determined using dynamic light scattering (DLS) with a Zetasizer Nano ZS90 (Malvern Panalytical Ltd., Malvern, United Kingdom). Prior to analysis, nectar samples were appropriately diluted with 0.22‐*μ*m‐filtered deionized water to minimize multiple scattering effects. The hydrodynamic particle diameter (*Z*‐average) was determined from the intensity‐weighted particle size distribution, whereas zeta potential was measured by electrophoretic light scattering using disposable folded capillary cells. All measurements were performed at 25^°^C ± 1^°^C after thermal equilibration, and each sample was analyzed in triplicate according to the manufacturer′s operating procedures. The results were expressed as mean ± SD.

### 2.8. Chemometric and Multivariate Analysis

Raman spectral data together with the physicochemical, antioxidant, bioactive compound, color, and particle characterization datasets were subjected to chemometric and multivariate statistical analyses to investigate treatment discrimination and the relationships among the measured quality attributes. Prior to chemometric analysis, Raman spectra were preprocessed by sequential cosmic‐ray removal, adaptive polynomial baseline correction, Butterworth smoothing, vector normalization, and peak alignment to minimize spectral noise and baseline variation while preserving characteristic Raman features [13, 14].

PCA was performed using mean‐centered and *Z*‐score standardized data to reduce data dimensionality, identify the major sources of variation, and visualize clustering patterns among the treatment conditions based on Raman molecular fingerprints and experimental quality characteristics. Pearson′s correlation coefficients (r) were calculated to evaluate the relationships among physicochemical properties, antioxidant activity, bioactive compounds, CIELAB color parameters, particle characteristics, and Raman spectral variables. The resulting correlation matrices were visualized using hierarchical correlation heatmaps and correlation network diagrams to facilitate the interpretation of variable associations.

To compare variables measured on different numerical scales, standardized *Z*‐score profiles were generated following autoscaling of the experimental dataset. In addition, integrated multivariate visualization maps were developed to illustrate the evolution of color characteristics and colloidal stability under the different ultrasound‐processing conditions. All chemometric analyses, statistical calculations, and graphical visualizations were performed using OriginPro 2024 (OriginLab Corporation, Northampton, Massachusetts, United States) and R software Version 4.3.2 (R Foundation for Statistical Computing, Vienna, Austria). The graphical outputs were generated using the ggplot2, corrplot, ComplexHeatmap, and factoextra packages implemented in the R environment.

### 2.9. Statistical Analysis

The experiment was conducted using a completely randomized design (CRD) with three independent replicates for each treatment. Data are presented as mean ± SD. Prior to statistical analysis, the assumptions of normality and homogeneity of variance were evaluated using the Shapiro–Wilk and Levene′s tests, respectively. Differences among treatment means were analyzed using one‐way analysis of variance (ANOVA), followed by Tukey′s honestly significant difference (HSD) multiple comparison test at a significance level of *p* < 0.05. The magnitude of treatment effects was quantified using partial eta‐squared ($\eta_{p}^{2}$) as a measure of effect size. Where appropriate, Holm–Bonferroni correction was applied to control the family‐wise Type I error associated with multiple comparisons. Statistical analyses were performed using IBM SPSS Statistics version 29.0 (IBM Corp., Armonk, New York, United States), whereas multivariate analyses and graphical visualizations were generated using OriginPro 2024 (OriginLab Corporation, Northampton, Massachusetts, United States) and R software Version 4.3.2 (R Foundation for Statistical Computing, Vienna, Austria).

## 3. Results

### 3.1. Characterization of the AgNP SERS Substrate

The citrate‐reduced AgNPs exhibited a characteristic surface‐plasmon‐resonance band at approximately 408 nm (Figure 1A), consistent with the ~400–420 nm region commonly reported for citrate‐reduced silver colloids and supporting successful nanoparticle formation [15, 23]. DLS analysis showed a *Z*‐average diameter of 42.6 ± 6.3 nm and a low PDI of 0.196 ± 0.031 (Figure 1B), indicating a relatively narrow hydrodynamic size distribution. Particle size and dispersity are important for SERS because nanoparticle dimensions and aggregation influence localized surface‐plasmon resonance and electromagnetic enhancement [13].

**Figure 1:**
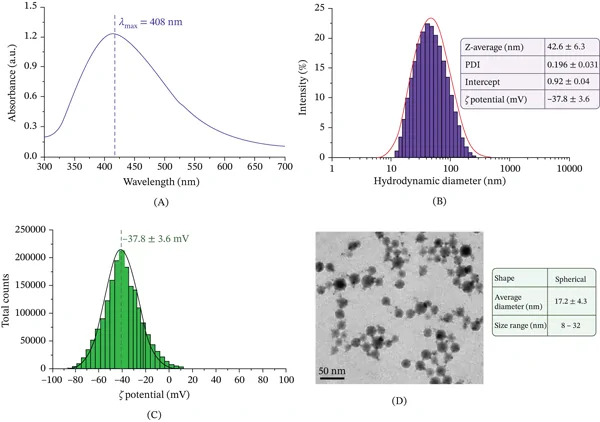
(A–D) Physicochemical and morphological characterization of citrate‐reduced AgNPs used as the SERS substrate.

The AgNP colloid exhibited a zeta potential of −37.8 ± 3.6 mV (Figure 1C). The negative surface charge is consistent with adsorption of citrate species on the AgNP surface, which promotes electrostatic repulsion and colloidal stabilization [15, 23]. Such stability is important for minimizing uncontrolled aggregation and improving reproducibility during SERS measurements [13].

Microscopy showed predominantly spherical nanoparticles, with a primary particle diameter of 17.2 ± 4.3 nm and an approximate range of 8–32 nm (Figure 1D). The smaller microscopy‐derived diameter compared with the DLS Z‐average is expected because microscopy measures the primary particle core, whereas DLS measures the effective hydrodynamic diameter of particles dispersed in liquid. Similar differences between microscopic and hydrodynamic nanoparticle dimensions are commonly observed in colloidal systems.

Overall, the 408‐nm plasmon band, low PDI, negative zeta potential, and predominantly spherical morphology support the colloidal quality of the AgNP substrate used for SERS. Because SERS enhancement depends strongly on nanoparticle size, morphology, surface chemistry, and aggregation state, this characterization strengthens the reproducibility and interpretation of the subsequent spectral analysis [13, 14].

### 3.2. SERS Molecular Fingerprint and Relationship With Bioactive Characteristics

The SERS spectra of stevia‐sweetened aguaymanto nectar exhibited four prominent bands at approximately 495, 1007, 1388, and 1613 cm^−1^ (Figure 2A). Their tentative vibrational assignments and plausible molecular contributors are summarized in Table 2. The band at 495 cm^−1^ was tentatively associated with low‐frequency skeletal/deformation vibrations of oxygenated structures, whereas the 1007 cm^−1^ band was primarily associated with C–C skeletal stretching. Notably, Raman analysis of purified steviol glycosides has identified C–C stretching of the steviol aglycone within 1004–1075 cm^−1^, supporting a possible contribution of steviol‐glycoside structures to the 1007 cm^−1^ signal [24].

**Figure 2:**
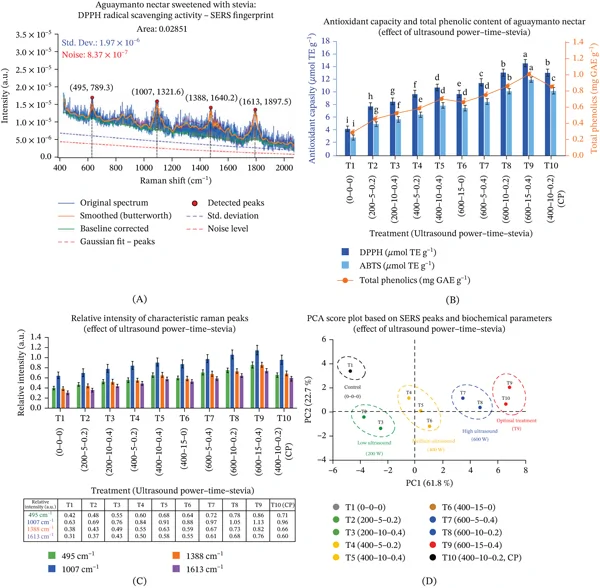
Integrated SERS molecular fingerprinting and chemometric characterization of stevia‐sweetened aguaymanto (*Physalis peruviana* L.) nectar under the evaluated processing‐formulation conditions. (A) Representative preprocessed SERS spectra showing the characteristic bands at approximately 495, 1007, 1388, and 1613 cm^−1^; (B) antioxidant and total phenolic characteristics; (C) relative intensities of the four characteristic SERS bands; and (D) PCA score plot based on the integrated spectral and biochemical profiles. Quantitative values are presented as mean ± SD (*n* = 3). Different lowercase letters indicate significant differences among treatment combinations according to one‐way ANOVA followed by Tukey′s HSD test (*p* < 0.05).

**Table 2:** Vibrational assignments and plausible molecular contributors of the principal SERS bands detected in stevia‐sweetened aguaymanto nectar.

SERS band (cm^−1^)	Tentative vibrational assignment	Plausible molecular contributors	Interpretation in the present matrix
495	Low‐frequency skeletal/ring deformation; possible C–C–O‐associated deformation	Oxygenated phytochemicals and carbohydrate/glycosidic structures	Collective low‐frequency fingerprint of oxygen‐rich constituents; not considered compound‐specific
1007	C–C skeletal/ring stretching	Steviol‐glycoside/aglycone structures; overlapping carbohydrate/glycosidic constituents	Compatible with the reported ~1004–1075 cm^−1^ C–C stretching region of the steviol aglycone
1388	C–H deformation and overlapping vibrations of oxygenated/phenolic structures	Phenolic, flavonoid, and glycosidic constituents	Represents an overlapping phytochemical fingerprint and should not be attributed to a single compound
1613	Aromatic/conjugated C=C skeletal stretching	Phenolic and flavonoid structures; possible hydroxycinnamic‐acid–related constituents	Consistent with aromatic/conjugated phenolic structures; compound‐specific identification requires authentic standards or chromatography

*Note*: Tentative assignments were based on Pezzotti et al. [24], Wang et al. [14], and Bahlol et al. [13]. Assignments represent plausible molecular‐class contributions rather than compound‐specific identification.

The 1388 cm^−1^ band was tentatively attributed to overlapping C–H deformation and oxygenated/phenolic‐associated vibrations, whereas the 1613 cm^−1^ band was associated predominantly with aromatic/conjugated C=C skeletal vibrations characteristic of phenolic structures. Because SERS spectra of complex fruit matrices contain overlapping contributions from multiple constituents and are affected by adsorption and molecular orientation at the AgNP surface, these assignments should be considered tentative molecular‐class associations rather than compound‐specific identification [13, 14].

Relative intensities of the characteristic bands varied among treatments, with pronounced responses around 1007 and 1613 cm^−1^ (Figure 2B,C). These spectral changes occurred concurrently with differences in phenolic content and antioxidant indices, supporting an association between the SERS fingerprint and the biochemical characteristics of the nectar. However, SERS intensity is influenced not only by analyte concentration but also by analyte–substrate interactions and electromagnetic enhancement; therefore, the observed intensity changes were interpreted as modifications of the overall molecular fingerprint rather than direct quantitative changes in individual compounds [13, 14].

PCA further differentiated the treatment combinations based on their integrated spectral and biochemical profiles (Figure 2D), with T9 clearly separated from the untreated control. Because ultrasound power, treatment duration, and stevia concentration were varied concurrently, this separation represents a combined treatment effect and cannot be attributed independently to ultrasound or stevia.

### 3.3. Physicochemical Properties of Ultrasound‐Assisted Stevia‐Sweetened Aguaymanto Nectar

Significant differences in physicochemical characteristics were observed among the evaluated processing‐formulation combinations (Figure 3). The pH ranged from 3.55 in T1 to 3.69 in T9, whereas total soluble solids decreased from 12.0 to 11.4°Brix and TA from 0.74 to 0.66%. Apparent viscosity ranged from 18.4 to 30.4 mPa·s, whereas turbidity decreased from 385 to 289 NTU. Among the evaluated conditions, T9 (600 W, 15 min, 0.4% stevia) exhibited the highest apparent viscosity and lowest turbidity, whereas the intermediate treatment T10 showed intermediate responses.

**Figure 3:**
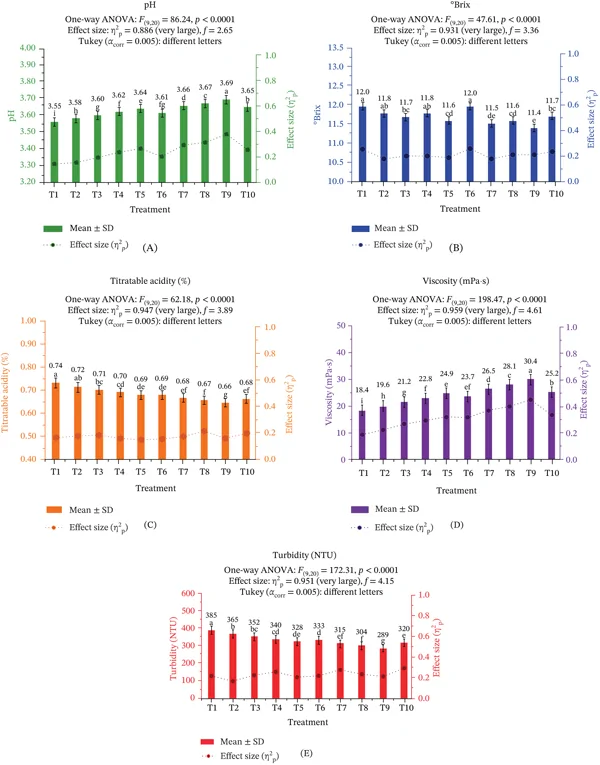
Physicochemical characteristics of stevia‐sweetened aguaymanto (*Physalis peruviana* L.) nectar under the evaluated processing‐formulation conditions. (A) pH; (B) total soluble solids; (C) titratable acidity; (D) apparent viscosity; and (E) turbidity. Values are presented as mean ± SD (*n* = 3). Different lowercase letters indicate significant differences among treatment combinations according to one‐way ANOVA followed by Tukey′s HSD test (*p* < 0.05).

One‐way ANOVA indicated significant differences among treatment combinations for all physicochemical variables (*p* < 0.0001), with pairwise differences identified by Tukey′s HSD test. The observed changes are consistent with cavitation‐associated particle disruption, enhanced dispersion, and improved homogenization reported for ultrasound‐treated plant matrices [8, 9]. Cavitation may also increase the accessibility of pectic and other soluble macromolecular constituents, potentially contributing to the observed viscosity differences. Similarly, changes in organic‐acid distribution or stability may contribute to the relatively small variations in pH and TA.

The large treatment effect sizes ($\eta_{p}^{2}>0.88$) indicate substantial differences among the predefined processing‐formulation conditions. However, because ultrasound power, treatment duration, and stevia concentration were varied concurrently, these effects cannot be assigned independently to any single processing variable. Accordingly, the results demonstrate differences among the complete treatment combinations rather than independent effects of ultrasound intensity.

### 3.4. Antioxidant Activity and Bioactive Compound Profile

Significant differences in antioxidant and phytochemical characteristics were observed among the evaluated processing‐formulation combinations (Figure 4A–F). DPPH radical‐scavenging capacity ranged from 5.2 to 11.6 *μ*mol TE g^−1^, ABTS from 7.8 to 16.9 *μ*mol TE g^−1^, and FRAP from 9.4 to 21.4 *μ*mol Fe^2+^ g^−1^. TPC ranged from 0.51 to 1.03 mg GAE g^−1^, whereas TFC ranged from 0.24 to 0.55 mg QE g^−1^. Among the evaluated conditions, T9 (600 W, 15 min, 0.4% stevia) exhibited the highest values for these antioxidant and phytochemical indices. The observed differences are consistent with reports that acoustic cavitation can disrupt residual plant structures, improve mass transfer, and increase the accessibility of intracellular phytochemicals in plant‐derived matrices [8, 9, 12]. These results should nevertheless be interpreted as responses to the complete processing‐formulation combinations because ultrasound power, treatment duration, and stevia concentration were varied concurrently.

**Figure 4:**
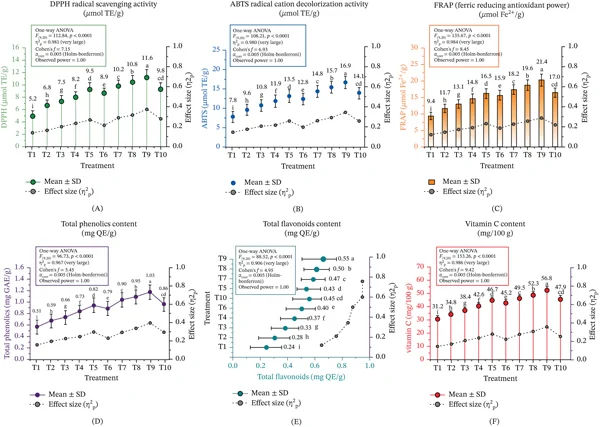
Antioxidant activity and bioactive‐compound characteristics of stevia‐sweetened aguaymanto (*Physalis peruviana* L.) nectar under the evaluated processing‐formulation conditions. (A) DPPH radical‐scavenging capacity; (B) ABTS radical‐cation scavenging capacity; (C) ferric reducing antioxidant power (FRAP); (D) total phenolic content; (E) total flavonoid content; and (F) DCPIP‐determined vitamin C. Values are presented as mean ± SD (*n* = 3). Different lowercase letters indicate significant differences among treatment combinations according to one‐way ANOVA followed by Tukey′s HSD test (*p* < 0.05).

DCPIP‐determined vitamin C increased from 31.2 mg/100 g in T1 to 56.8 mg/100 g in T9, corresponding to an approximately 82% higher observed value. Although cavitation can enhance the release and accessibility of soluble intracellular constituents [8, 9], the nectar had already undergone high‐shear homogenization at 10,000 rpm for 2 min before sonication. Therefore, the magnitude of this difference cannot be attributed solely to additional release of intracellular ascorbic acid during ultrasound treatment. Furthermore, because DCPIP is a redox‐based method without chromatographic separation of L‐ascorbic acid, potential contributions from other reducing constituents in the nectar matrix cannot be excluded. The reported values are therefore retained as experimentally observed DCPIP‐determined vitamin C values, whereas confirmation of the magnitude of the compound‐specific change in L‐ascorbic acid would require a selective chromatographic method such as HPLC‐DAD.

The coordinated increases in DPPH, ABTS, FRAP, TPC, and TFC indicate substantial differences in the overall antioxidant characteristics of the treatment combinations. Cavitation‐associated shear forces, microstreaming, and turbulence provide a plausible mechanism for enhanced accessibility of phenolic and other soluble constituents [8, 9], whereas ultrasound‐assisted processing has been associated with improved extraction efficiency of bioactive compounds in plant matrices [12]. Stevia may also have contributed to the observed antioxidant response because *S*. *rebaudiana* contains phenolic and other antioxidant‐active constituents [6, 7].

Accordingly, the superior antioxidant and phytochemical profile of T9 should be interpreted as the response of the combined processing‐formulation condition, rather than as an independent effect of high ultrasound power. The relative contributions of ultrasound power, treatment duration, and stevia concentration cannot be separated using the present experimental design. Future factorial studies incorporating matched ultrasound‐ and stevia‐only controls, together with chromatographic determination of L‐ascorbic acid, would enable more definitive interpretation of the individual processing and compositional effects.

### 3.5. CIELAB Color Characteristics and Multivariate Color Analysis

Ultrasound processing significantly modified the CIELAB color characteristics of aguaymanto nectar. The three‐dimensional CIELAB trajectory demonstrated a progressive shift in color coordinates from the control toward the more intensively treated samples (Figure 5A). The response‐surface visualization further showed the distribution of lightness across the treatment space, with the best performing treatment occupying the region associated with enhanced color characteristics (Figure 5B). Standardized color profiles showed coordinated changes in L*, a*, b*, and *Δ*E_00_ across the selected treatments (Figure 5C). PCA explained 99.8% of the total variance (PC1 = 96.2*%*; PC2 = 3.6*%*) and clearly separated the treatments according to their overall color characteristics (Figure 5D). The individual color metrics further demonstrated treatment‐dependent changes in chroma, hue angle, WI, BI, and YI (Figure 5E). Finally, the correlation network revealed strong relationships among the color parameters, including positive associations among b*, chroma, yellowness‐related characteristics, and *Δ*E_00_ (Figure 5F).

**Figure 5:**
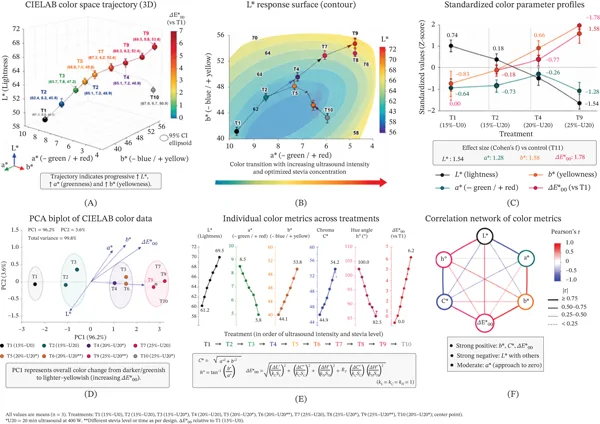
Comprehensive multivariate evaluation of the CIELAB color characteristics of ultrasound‐assisted stevia‐sweetened aguaymanto (*Physalis peruviana* L.) nectar. (A) CIELAB color space trajectory (3D); (B) L* response surface (contour); (C) standardized color parameter profiles; (D) PCA biplot of CIELAB color data; (E) individual color metrics across treatments; (F) correlation network of color metrics.

The observed color modifications can be attributed to the physical effects of acoustic cavitation, which improves particle dispersion, reduces suspended particle size, and promotes a more homogeneous distribution of carotenoid pigments within the nectar matrix [8, 9]. Improved light scattering caused by smaller particles increases lightness, whereas enhanced extraction and stabilization of carotenoids contribute to the higher yellowness values. The reduction in a* suggests a shift away from reddish tones, possibly due to pigment redistribution and partial degradation of red‐colored compounds during sonication. The PCA and correlation analyses confirmed that ultrasound treatment consistently altered the overall color profile rather than individual parameters independently, indicating coordinated physicochemical changes within the beverage system. These findings demonstrate that combining CIELAB colorimetry with multivariate statistical analysis provides a robust approach for monitoring color quality and identifying optimal processing conditions in functional fruit beverages.

### 3.6. Particle Characteristics and Colloidal Stability

The particle characterization demonstrated that ultrasound processing markedly improved the colloidal properties of aguaymanto nectar. Particle size decreased progressively from 980 (T1) to 270 nm (T9), corresponding to a reduction of approximately 72.4%, whereas the absolute zeta potential increased from −18.2 to −37.8 mV, and the PDI decreased from 0.49 to 0.21 (Figure 6A). The integrated colloidal‐stability domain map clearly illustrated the transition of treatments from the unstable region toward the highly stable region across the progressively more intensive processing‐formulation conditions evaluated (Figure 6B). Likewise, the normalized multidimensional particle profile demonstrated progressive improvements in particle uniformity and dispersion characteristics across the treatments (Figure 6C), whereas the distribution signatures provided a complementary visualization of changes in particle size, zeta potential, and PDI (Figure 6D). The stability‐evolution pathway further identified T9 as the treatment exhibiting the greatest colloidal stability (Figure 6E). Finally, the parameter‐interrelationship matrix revealed a strong negative relationship between particle size and zeta potential and a positive association between particle size and PDI (Figure 6F), confirming that particle‐size reduction was accompanied by enhanced surface charge and improved homogeneity dispersion. Although acoustic cavitation provides a plausible mechanism for particle disruption and improved dispersion, the present experimental design does not permit the magnitude of the ultrasound contribution to be separated statistically from treatment time or stevia concentration. Therefore, the observed colloidal improvements should be interpreted as responses to the complete treatment combinations.

**Figure 6:**
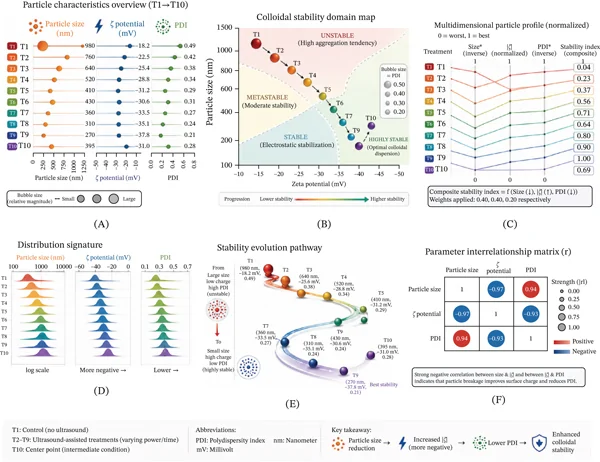
Integrated characterization of particle properties and colloidal stability of ultrasound‐assisted stevia‐sweetened aguaymanto (*Physalis peruviana* L.) nectar: (A) particle‐characteristics overview; (B) colloidal‐stability domain map; (C) normalized multidimensional particle profile; (D) distribution signatures; (E) stability‐evolution pathway; and (F) interrelationship matrix of particle‐size, zeta‐potential, and PDI parameters.

Acoustic cavitation provides a plausible mechanism contributing to the observed colloidal differences. Cavitation‐bubble formation and collapse generate localized shear forces, microjets, and turbulence capable of disrupting suspended particle aggregates [8, 25]. Smaller particles may provide greater interfacial area for adsorption of charged constituents, while increased absolute zeta potential can enhance electrostatic repulsion and restrict aggregation [9]. Similar particle‐size reductions and improvements in dispersion characteristics have been reported in ultrasound‐treated fruit and plant‐based systems [12].

#### 3.6.1. Limitations and Prospects

Several limitations should be considered when interpreting the present findings. First, ultrasound power, treatment duration, and stevia concentration were varied concurrently across the 10 predefined processing‐formulation conditions rather than within a factorial or response‐surface design. Consequently, the independent main effects and interactions of these variables cannot be estimated from the present dataset. Although T9 (600 W, 15 min, 0.4% stevia) exhibited the most favorable overall instrumental profile, its performance cannot be attributed exclusively to ultrasound intensity, treatment duration, or stevia concentration. The present experiment should therefore be regarded as an exploratory comparison of combined treatment conditions rather than a statistical optimization study. Future studies should employ factorial, Box–Behnken, or central composite designs incorporating matched ultrasound‐ and stevia‐only controls to quantify individual and interactive effects.

Second, vitamin C was experimentally determined using the DCPIP redox method rather than a compound‐selective chromatographic technique. The reported values therefore represent observed DCPIP‐determined vitamin C measurements; however, potential contributions from other reducing constituents in the fruit matrix cannot be excluded, particularly because phenolic and flavonoid content also varied among treatments. Consequently, the higher value observed for T9 should not be interpreted as definitive evidence of an equivalent compound‐specific increase in L‐ascorbic acid. Future studies should validate L‐ascorbic acid and, where relevant, dehydroascorbic acid using HPLC‐DAD or LC‐MS.

Third, microbiological inactivation and storage stability were not evaluated. Although high‐intensity ultrasound can induce microbial injury through cavitation‐associated shear forces, pressure gradients, and localized physicochemical effects, antimicrobial efficacy depends strongly on treatment conditions and food‐matrix characteristics [8, 9, 11]. Therefore, T9 should not be considered microbiologically equivalent to thermal pasteurization or sufficient to ensure beverage safety based on the present findings. Microbial challenge testing, total viable counts, yeast and mold enumeration, and controlled refrigerated‐storage studies are required to establish microbial efficacy and shelf life.

Sensory acceptability was not assessed. This limitation is particularly relevant to T9 because it contained the highest stevia concentration (0.4%). Steviol glycosides may produce bitter, metallic, or lingering aftertaste depending on concentration, glycoside composition, food matrix, and consumer sensitivity [6, 7]. Therefore, the favorable instrumental characteristics of T9 do not necessarily indicate consumer preference. Future optimization should integrate sensory and consumer‐acceptance testing with chromatographic validation, microbiological safety, storage stability, and process scale‐up before commercial application.

#### 3.6.2. Industrial Implications

The present findings indicate that ultrasound‐assisted processing warrants further investigation as a nonthermal processing strategy for stevia‐sweetened aguaymanto beverages. Among the evaluated conditions, T9 (600 W, 15 min, 0.4% stevia) produced the most favorable immediate physicochemical, antioxidant, color, and colloidal characteristics. However, these instrumental improvements alone are insufficient to establish an industrially optimal formulation because microbiological safety, shelf‐life stability, and sensory acceptability were not evaluated.

Particular consideration should be given to the relatively high stevia concentration used in T9. Although stevia can support reduced‐sugar beverage development, excessive sweetness or bitter, metallic, and lingering aftertaste may limit consumer acceptance [6, 7]. Therefore, future optimization should balance physicochemical and antioxidant performance against sensory responses rather than selecting a processing condition exclusively from instrumental quality measurements.

Scale‐up also requires validation of ultrasound energy delivery under larger processing volumes. Acoustic energy distribution, cavitation intensity, treatment uniformity, temperature control, energy consumption, and throughput may differ substantially between laboratory probe sonication and continuous industrial systems. Furthermore, microbial lethality must be established before ultrasound can be considered a preservation treatment or alternative to conventional pasteurization. Ultrasound may ultimately be more appropriate as part of a combined preservation strategy, depending on the microbial reduction and shelf‐life requirements of the final beverage. Integration of SERS with conventional analytical measurements may nevertheless provide a useful platform for rapid monitoring of molecular and quality changes during subsequent process‐development studies.

## 4. Conclusions

Significant differences in the molecular, physicochemical, antioxidant, color, and colloidal characteristics of stevia‐sweetened aguaymanto (*P*. *peruviana* L.) nectar were observed among the evaluated processing‐formulation conditions. Among the 10 treatments, T9 (600 W, 15 min, 0.4% stevia) exhibited the most favorable overall instrumental profile, including the highest antioxidant, phenolic, flavonoid, and DCPIP‐determined vitamin C values, together with favorable physicochemical, color, and colloidal characteristics. Particle size decreased from 980 to 270 nm, the absolute zeta potential increased from −18.2 to −37.8 mV, and PDI decreased from 0.49 to 0.21.

SERS fingerprinting revealed treatment‐dependent differences in characteristic bands at approximately 495, 1007, 1388, and 1613 cm^−1^, whereas chemometric analysis differentiated the treatment combinations according to their integrated molecular and biochemical profiles. These findings support the potential of SERS as a complementary approach for rapid comparative assessment of quality changes in functional fruit beverages. The spectral assignments, however, represent tentative molecular‐class associations rather than definitive identification of individual compounds.

The observed DCPIP‐determined vitamin C content increased from 31.2 mg/100 g in T1 to 56.8 mg/100 g in T9. Because the DCPIP assay is based on a redox reaction and lacks chromatographic specificity for L‐ascorbic acid, potential contributions from other reducing constituents cannot be excluded. Thus, the experimentally observed values are retained, whereas their interpretation as a compound‐specific increase in L‐ascorbic acid requires chromatographic confirmation. Furthermore, because ultrasound power, treatment duration, and stevia concentration were varied concurrently, the superior performance of T9 cannot be attributed independently to any single factor. T9 should therefore be considered the best performing combined condition among those evaluated rather than a statistically optimized formulation. Future studies incorporating factorial optimization, chromatographic validation, microbial and storage assessment, and sensory evaluation are required before industrial application.

## Author Contributions

Betzabeth Brígida Huamán Estrada contributed to the conceptualization, methodology, investigation, formal analysis, data curation, visualization, writing of the original draft, and project administration. Franklin Oré Areche contributed to the methodology, investigation, validation, formal analysis, and manuscript review and editing. Peter Llimpe Perez contributed to the investigation, data curation, validation, resource provision, and manuscript review and editing. Norma Elvira Muguruza Crispin contributed to the formal analysis, software, visualization, and manuscript review and editing. Jorge Luis Rojas Paz contributed to the software, statistical analysis, data curation, and visualization. Fiorella Victoria Luperdi Rios contributed to the investigation, validation, resource provision, and manuscript review and editing. Jacqueline Roxana Reaño Rivera contributed to the methodology, formal analysis, validation, and manuscript review and editing. Tania Jakeline Choque Rivera contributed to the investigation, data curation, validation, and resource provision. Genaro Christian Pesantes Arriola contributed to the supervision, project administration, resource provision, and manuscript review and editing. Carmen del Pilar Alvarez Quinteros contributed to conceptualization, supervision, project administration, and manuscript review and editing.

## Funding

No funding was received for this manuscript.

## Disclosure

All authors have read and approved the final version of the manuscript and agree to be accountable for all aspects of the work. The R scripts used for chemometric analysis, statistical calculations, and graphical visualization are available from the corresponding author upon reasonable request.

## Ethics Statement

This study did not involve human participants or experimental animals. Therefore, ethical approval was not required.

## Consent

The authors have nothing to report.

## Conflicts of Interest

The authors declare no conflicts of interest.

## Data Availability

The data that support the findings of this study are available on request from the corresponding author. The data are not publicly available due to privacy or ethical restrictions.

## References

[1] Ramos C. , Lezama-Asencio P. , Asmat P. , Larco-León E. , Chuna-Mogollón P. , and Hidalgo M. , Aguaymanto L.: Fitoquímicos y Aplicaciones en la Salud Humana, Revista Mexicana de Ciencias Agrícolas. (2025) 16, no. 5, e3682, 10.29312/remexca.v16i5.3682.

[2] García-Godos P. , Prado-Sumari I. , Estrada R. , and Millán B. , In Situ Morphological Characterization of 34 Accessions of Physalis peruviana L. From the Department of Ayacucho (Peru), Information Technology. (2022) 33, no. 2, 169–180.

[3] Ramadan M. F. , Bioactive Phytochemicals, Nutritional Value, and Functional Properties of Cape Gooseberry (Physalis peruviana): An Overview, Food Research International. (2011) 44, no. 7, 1830–1836, 10.1016/j.foodres.2010.12.042.

[4] Pérez-Lamela C. and Torrado-Agrasar A. M. , Effects of High-Pressure Processing (HPP) on Antioxidant Vitamins (A, C, and E) and Antioxidant Activity in Fruit and Vegetable Preparations: A Review, Applied Sciences. (2025) 15, no. 19, 10699, 10.3390/app151910699.

[5] Kumari A. , Biswas P. , and Kumar N. , Recent Advancement in Medicinal Properties and Safety Aspects of Stevia rebaudiana: Zero-calorie Sugar Plant, Sugar Tech. (2026) 28, no. 1, 1–21, 10.1007/s12355-025-01658-3.

[6] Ahmad J. , Khan I. , Blundell R. , Azzopardi J. , and Mahomoodally M. F. , Stevia rebaudiana Bertoni.: An Updated Review of Its Health Benefits, Industrial Applications and Safety, Trends in Food Science & Technology. (2020) 100, 177–189, 10.1016/j.tifs.2020.04.030.

[7] Samuel P. , Ayoob K. T. , Magnuson B. A. , Wölwer-Rieck U. , Jeppesen P. B. , Rogers P. J. , and Mathews R. , Stevia Leaf to Stevia Sweetener: Exploring Its Science, Benefits, and Future Potential, Journal of Nutrition. (2018) 148, no. 7, 1186S–1205S, 10.1093/jn/nxy102, 29982648. 29982648

[8] Ashokkumar M. , Applications of Ultrasound in Food and Bioprocessing, Ultrasonics Sonochemistry. (2015) 25, 17–23, 10.1016/j.ultsonch.2014.08.012. 25219872

[9] Chemat F. , Rombaut N. , Sicaire A. G. , Meullemiestre A. , Fabiano-Tixier A. S. , and Abert-Vian M. , Ultrasound Assisted Extraction of Food and Natural Products. Mechanisms, Techniques, Combinations, Protocols and Applications: A review, Ultrasonics Sonochemistry. (2017) 34, 540–560, 10.1016/j.ultsonch.2016.06.035, 27773280. 27773280

[10] Mgoma S. T. , Basitere M. , Mshayisa V. V. , and De Jager D. , A Systematic Review on Sustainable Extraction, Preservation, and Enhancement in Food Processing: The Advancement From Conventional to Green Technology Through Ultrasound, Processes. (2025) 13, no. 4, 10.3390/pr13040965.

[11] Sotelo-Lara D. M. , Amador-Espejo G. G. , Álvarez-Araiza D. F. , Cordero-Rivera A. K. , Millán-Quintero K. G. , Campos-Vega R. , and Velázquez-Estrada R. M. , Ultrasound and Thermosonication as Promising Technologies for Processing Plant-Based Beverages: A Review, Food Technology and Biotechnology. (2024) 62, no. 4, 538–552, 10.17113/ftb.62.04.24.8624, 39830872. PMC11740745 39830872

[12] Tiwari B. K. , Ultrasound: A Clean, Green Extraction Technology, TrAC Trends in Analytical Chemistry. (2015) 71, 100–109, 10.1016/j.trac.2015.04.013.

[13] Bahlol H. S. , Li J. , Deng J. , Foda M. F. , and Han H. , Recent Progress in Nanomaterial-Based Surface-Enhanced Raman Spectroscopy for Food Safety Detection, Nanomaterials. (2024) 14, no. 21, 10.3390/nano14211750, 39513830. PMC11547707 39513830

[14] Wang J. , Chen Q. , Belwal T. , Lin X. , and Luo Z. , Insights Into Chemometric Algorithms for Quality Attributes and Hazards Detection in Foodstuffs Using Raman/Surface Enhanced Raman Spectroscopy, Comprehensive Reviews in Food Science and Food Safety. (2021) 20, no. 3, 2476–2507, 10.1111/1541-4337.12741, 33884762. 33884762

[15] Lee P. C. and Meisel D. , Adsorption and Surface-Enhanced Raman of Dyes on Silver and Gold Sols, Journal of Physical Chemistry. (1982) 86, no. 17, 3391–3395, 10.1021/j100214a025.

[16] AOAC International , Official Methods of Analysis, 2023, 22nd edition, AOAC International.

[17] Gulcin İ. and Alwasel S. H. , DPPH Radical Scavenging Assay, Processes. (2023) 11, no. 8, 10.3390/pr11082248.

[18] Re R. , Pellegrini N. , Proteggente A. , Pannala A. , Yang M. , and Rice-Evans C. , Antioxidant Activity Applying an Improved ABTS Radical Cation Decolorization Assay, Free Radical Biology and Medicine. (1999) 26, no. 9-10, 1231–1237, 10.1016/S0891-5849(98)00315-3, 10381194. 10381194

[19] Benzie I. F. and Strain J. J. , The Ferric Reducing Ability of Plasma (FRAP) as a Measure of “Antioxidant Power”: The FRAP Assay, Analytical Biochemistry. (1996) 239, no. 1, 70–76, 10.1006/abio.1996.0292, 8660627. 8660627

[20] Singleton V. L. , Orthofer R. , and Lamuela-Raventós R. M. , [14] Analysis of Total Phenols and Other Oxidation Substrates and Antioxidants by Means of Folin-ciocalteu Reagent, Methods in Enzymology, 1999, 299, Academic Press, 152–178, 10.1016/S0076-6879(99)99017-1.

[21] Chang C. C. , Yang M. H. , Wen H. M. , and Chern J. C. , Estimation of Total Flavonoid Content in Propolis by Two Complementary Colorimetric Methods, Journal of Food and Drug Analysis. (2002) 10, no. 3.

[22] Pathare P. B. , Opara U. L. , and Al-Said F. A. J. , Colour Measurement and Analysis in Fresh and Processed Foods: A Review, Food and Bioprocess Technology. (2013) 6, no. 1, 36–60, 10.1007/s11947-012-0867-9. PMC7089433 32215166

[23] Dong X. , Ji X. , Wu H. , Zhao L. , Li J. , and Yang W. , Shape Control of Silver Nanoparticles by Stepwise Citrate Reduction, Journal of Physical Chemistry C. (2009) 113, no. 16, 6573–6576, 10.1021/jp900775b.

[24] Pezzotti G. , Zhu W. , Aoki T. , Miyamoto A. , Fujita I. , Nakagawa M. , and Kobayashi T. , Raman Spectroscopic Analysis of Steviol Glycosides: Spectral Database and Quality Control Algorithms, Foods. (2024) 13, no. 19, 10.3390/foods13193068, 39410103. PMC11476355 39410103

[25] Kentish S. and Ashokkumar M. , The Physical and Chemical Effects of Ultrasound, Ultrasound Technologies for Food and Bioprocessing, 2010, Springer New York, 1–12.

